# Development of a Multispectral Albedometer and Deployment on an Unmanned Aircraft for Evaluating Satellite Retrieved Surface Reflectance over Nevada’s Black Rock Desert

**DOI:** 10.3390/s18103504

**Published:** 2018-10-17

**Authors:** Jayne M. Boehmler, S. Marcela Loría-Salazar, Chris Stevens, James D. Long, Adam C. Watts, Heather A. Holmes, James C. Barnard, W. Patrick Arnott

**Affiliations:** 1 Atmospheric Sciences Program, Department of Physics, University of Nevada, Reno, NV 89557, USA, j.boehmler915@gmail.com (J.M.B.); marce.marcelaloria@gmail.com (S.M.L.-S.); c.stevens@nevada.unr.edu (C.S.); jameslong@unr.edu (J.D.L.); hholmes@unr.edu (H.A.H.); jbarnard@unr.edu (J.C.B.); 2 Division of Atmospheric Sciences, Desert Research Institute, Reno, NV 89512, USA; Adam.Watts@dri.edu

**Keywords:** albedo, *AOD*, MODIS, LANDSAT, unmanned aircraft system, UAS, UAV, drone, satellite remote sensing

## Abstract

Bright surfaces across the western U.S. lead to uncertainties in satellite derived aerosol optical depth (*AOD*) where *AOD* is typically overestimated. With this in mind, a compact and portable instrument was developed to measure surface albedo on an unmanned aircraft system (UAS). This spectral albedometer uses two Hamamatsu micro-spectrometers (range: 340–780 nm) for measuring incident and reflected solar radiation at the surface. The instrument was deployed on 5 October 2017 in Nevada’s Black Rock Desert (BRD) to investigate a region of known high surface reflectance for comparison with albedo products from satellites. It was found that satellite retrievals underestimate surface reflectance compared to the UAS mounted albedometer. To highlight the importance of surface reflectance on the *AOD* from satellite retrieval algorithms, a 1-D radiative transfer model was used. The simple model was used to determine the sensitivity of *AOD* with respect to the change in albedo and indicates a large sensitivity of *AOD* retrievals to surface reflectance for certain combinations of surface albedo and aerosol optical properties. This demonstrates the need to increase the number of surface albedo measurements and an intensive evaluation of albedo satellite retrievals to improve satellite-derived *AOD*. The portable instrument is suitable for other applications as well.

## 1. Introduction

Atmospheric processes are driven by the global distribution of solar energy absorbed and reflected by the earth’s surface. The amount of energy that the earth absorbs or reflects over a given area depends on surface cover. Albedo, an important driver of the earth’s climate system, is a measure of surface reflectivity. The earth’s radiative balance can be affected by changes in albedo such as those due to land use change, deforestation, fires, snow, and ice cover. The earth’s average global albedo is being affected by anthropogenic activities such as urbanization, and the presence of aerosols in the atmosphere which can be deposited onto snow [1]. Accurate measurements of surface albedo are needed for understanding the climatological ramifications of land use change, reducing uncertainties in global climate models [2] and improving satellite retrievals of aerosol optical depth (*AOD*) [3]. However, the effects of albedo changes on global radiative forcing are still highly uncertain due to the wide range of estimates of anthropogenic and natural land cover change [4]. More comprehensive methods for accurately measuring regional albedo over time are needed.

Quantifying albedo is complex because it varies in both space and time and it is highly dependent on solar zenith angle [5]. It is a dimensionless quantity that can be defined as the ratio of the solar irradiance reflected from the earth’s surface to that which is incident upon it [6]. Broadband albedo ground measuring devices, such as pyranometers, are widely used in the field yet only provide a single measurement integrated across a wide spectral range. Global networks, such as the National Oceanic and Atmospheric Administration (NOAA) Surface Radiation network and the Department of Energy (DOE) Atmospheric Radiation Measurement network utilize broadband pyranometers and narrowband radiometers on fixed towers for measuring albedo but are limited by their spatial footprint.

Satellite characterization of surface albedo is desired due to the limited direct measurements at the surface. While satellite retrievals of albedo provide global coverage, accurately estimating albedo from space-borne platforms can be challenging because of the spatial-temporal variability of the surface and atmosphere. Previous studies have evaluated satellite surface albedo using ground-based networks as well as inter-comparisons between space-borne instruments [7,8,9]. Generally, satellite and ground-based measurements of albedo agree over vegetated landscapes [10,11,12]. However, variable and uncertain surface albedo measurements have resulted in many global satellite aerosol products being limited to ocean retrievals exclusively [13].

Aerosol remote sensing is subject to four primary factors that can cause uncertainty: (1) sensor calibration, (2) cloud screening, (3) intensive and extensive aerosol optical properties parametrizations, and (4) surface reflectance characterization [13]. Acquiring satellite-derived measurements with high accuracy can be especially challenging over regions of complex terrain as well as in arid or semi-arid environments (e.g., the western US) [14,15]. Previous studies have found that satellite retrievals of *AOD* can be overestimated when the underlying surface has high reflectance or shadowing effects due to topography [16]. In current *AOD* products from the Moderate Resolution Imaging Spectroradiometer (MODIS), this is designated by a quality flag and the pixels are removed from the *AOD* dataset (e.g., Black Rock Desert, NV and Rogers Dry Lake, CA, USA) [14]. Moreover, it has been reported that an error of 0.01 in estimated surface reflectance can translate to an error of 0.1 in satellite derived *AOD* [3].

Unmanned Aircraft Systems (UAS) and other small aircraft observations have the potential to provide cost-effective, low-altitude measurements for atmospheric science applications. They can provide platforms for optical observations of the surface with greater accuracy than conventional high-altitude satellites and manned airplanes due to the reduced effect of atmospheric extinction and higher spatial resolution [17]. Although albedometers have been previously mounted onto small planes [18,19], fewer studies to measure albedo from UAS have been performed. Uto et al. (2016) [17] developed a low-cost hyperspectral whiskbroom imager for UAS applications using Hamamatsu micro-spectrometers. Previous work to develop albedometers has consisted mainly of long poles with a commercial pyranometer or spectrometer attached to the top end to be used for surveying on foot [20]. This technique is inconveniently heavy and often requires extra equipment to operate and log data.

The present work, in part, addresses the need for more portable ground-based measurements for evaluating satellite retrievals over areas of known high surface reflectance through the development of a novel multispectral albedometer for measuring hemispherical albedo on a UAS. One aim of this paper is to evaluate satellite retrievals of albedo from the MODIS twin instruments on board the National Aeronautics and Space Administration (NASA) Terra and Aqua satellites as well as the Enhanced Thematic Mapper Plus instrument (ETM+) on board Land Satellite 7 (LANDSAT7) over complex, semi-arid desert terrain against the developed albedometer onboard the UAS. The effect of surface reflectance on *AOD* from the MODIS deep-blue algorithm is also discussed. To summarize, the goals of this paper are threefold: (1) develop a small portable albedometer, (2) evaluate the portable instrument using satellite retrievals, and (3) investigate the impact of surface reflectance on satellite *AOD*. The following section details the sensor design and specifications, Section 3 describes the UAS field experiment and satellite remote sensing products, Section 4 presents the results from Nevada’s Black Rock Desert (BRD) compared to satellite products in October 2017, and Section 5 discusses the comparison to satellite-retrieved values as well as future developments and applications for the instrument.

## 2. Instrument Design and Testing

**Figure 1:**
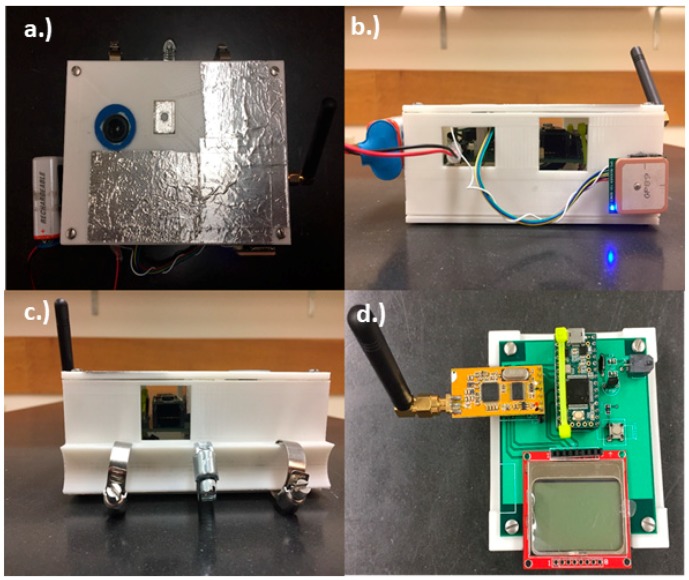
Albedometer design: (**a**) Top view of measuring device showing upward facing spectrometer and camera. Aluminum tape was added to maintain cool temperatures inside the box and an ultraviolet (UV)/infra-red (IR) filter was placed over the camera to capture more natural looking images; (**b**) Side view of measuring device showing the Global Positioning System (GPS) and 9 V battery which sit outside of the box; (**c**) Side view of measuring device showing the custom 3D-printed mount built-in to the box; (**d**) Ground control device showing radio for communicating to the measuring device, a button for initiating measurements, a screen for printing resulting albedo in real-time, and the Teensy 3.2 microcontroller.

The instrument consists of two parts. The first part is a measuring device mounted to the aircraft (~300 g), which houses two micro-spectrometers and six additional sensors, as shown in Figure 1a–c. The second part is a handheld display and ground control device (~133 g) to initiate collection and display real-time data from the measuring device, as shown in Figure 1d. The measuring device is enclosed in a 3D-printed polylactic acid (PLA) casing with a custom mount built-in to the design of the box, as shown in Figure 1. Both parts are powered by 9 V batteries and can operate for multiple hours. Teensy 3.6 and 3.2 microcontrollers are used to control signal processing for each part, respectively [21]. The Teensy 3.6 microcontroller has a built-in real-time clock with battery backup capability for time and date. To measure albedo, two micro-spectrometers manufactured by Hamamatsu Photonics, each with a spectral range of 340–780 nm, are utilized; one for obtaining the downwelling solar radiation and the other for measuring the solar radiation reflected from the surface. Albedo values range from 0 to 1 and are calculated as the ratio between reflected light from the surface (downward facing spectrometer) and incident light (upward facing spectrometer), shown in Equation (1).

(1) $$\;Albedo\;\left(\lambda \right)=\;\frac{Downward\;facing\;spectrometer}{Upward\;facing\;spectrometer}\;$$

The spectrometer values used to calculate albedo must include corrections to account for dark counts, integration time, and a transfer function that accounts for using two different sensors. All of these will be presented in Section 2.2 where the system calibration and testing are discussed.

The uncertainty for albedo measurements was also calculated with every measurement for each wavelength using Equation (2).

(2) $$\;Uncertainty\;\left(\lambda \right)=Albedo\times 0.5\times \sqrt{\frac{1}{Spec_{1}}+\frac{1}{Spec_{2}}}\;$$

*Spec*_1_ and *Spec*_2_ are the counts (with dark counts subtracted) from the upward facing and downward facing spectrometers, respectively. Equation (2) follows from the propagation of uncorrelated error and uncertainty in spectrometer counts as the square root of the counts.

**Figure 2:**
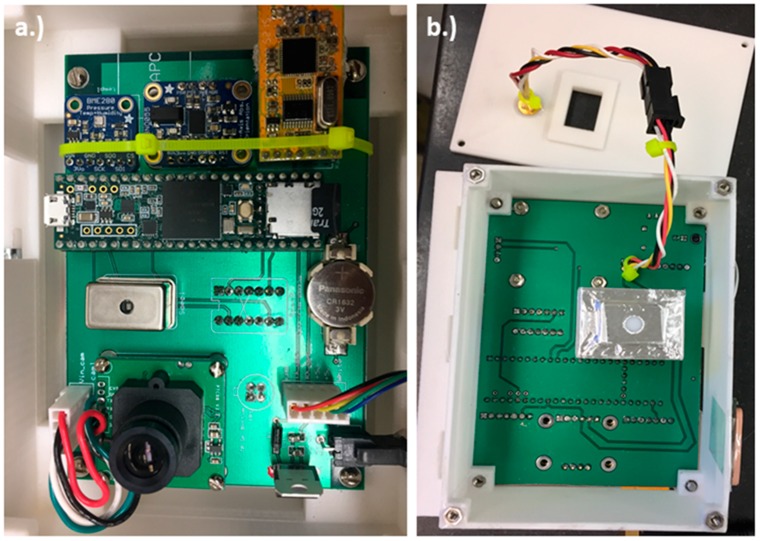
Albedometer components: (**a**) Top view of printed circuit board including components (from left to right, top to bottom): BME280 temperature sensor, BNO055 absolute orient, APC220 radio, Teensy 3.6 microcontroller, C12666MA micro-spectrometer, Back-up battery, VC0706 camera, UBX-G7020 GPS; (**b**) Bottom view of printed circuit board including the C12666MA spectrometer with diffuser and MLX90614 infra-red (IR) sensor.

Additional components on the instrument include a Global Positioning System (GPS) for position, altitude, and time; a digital level and compass for measuring instrument orientation; temperature, pressure, and humidity sensors; an infrared sensor to measure ground temperature; a camera for measuring sky conditions; a radio for two-way communication between the devices; and a micro secure digital (SD) card for recording data, as shown in Figure 2. Specific connections for all components in the design of the albedometer are shown in the circuit board schematic available at the following URL: http://www.patarnott.com/atms360/boards.html. Additionally, the 3-D printing files and the code used to run the instrument are provided at the same URL.

### 2.1. Sensor Components

#### 2.1.1. C12666MA Micro-Spectrometer 

The Hamamatsu micro-spectrometers used for obtaining albedo feature an ultra-compact design with size dimensions 20.1 × 12.5 × 10.1 mm and mass of 5 g. The manufacturer specifications indicate a spectral range from 340 to 780 nm and a spectral resolution of 15 nm. In this application we only considered 400 to 750 nm due to low counts below 400 nm. The micro-spectrometer employs a reflective concave blazed grating to diffract incident light entered through an optical slit. The diffracted beam is cast to a highly-sensitive complementary metal-oxide-semiconductor (CMOS) linear image sensor chip [22].

#### 2.1.2. Teensy 3.6/3.2 Microcontroller

A 32-bit, 180 MHz Advanced Reduced Instruction Set Computer Machine (ARM) processer controls the functionality of the system. The microcontroller performs analog to digital conversion with a 13-bit read resolution and is programmed using the Arduino Integrated Development Environment (IDE), an open-source software designed to easily write and upload code to Arduino compatible hardware [23].

#### 2.1.3. BME 280 Pressure, Temperature, and Humidity Sensor 

Digital readings of pressure, temperature, and humidity were obtained in conjunction with every observation. The Bosch sensor is able to measure conditions within the control box with a response time of 1 s and was incorporated into the instrument design using the inter-integrated circuit (I2C) interface. The pressure and temperature measurements were useful for determining the height of the UAS above the surface.

#### 2.1.4. BNO055 Absolute Orientation

The Bosch absolute orientation sensor was used to measure the tilt angle of the instrument relative to the vertical coordinate. The level reading was used as a data qualifier. Only measurements obtained when the aircraft was within a 5-degree offset in the *x*- and *y*-horizontal directions were used in our analysis. 

#### 2.1.5. VC0706 TTL Serial Camera

The onboard camera developed by Adafruit Industries was used to document the sky conditions at the time of measurement. An optical filter was used over the camera to block ultraviolet (UV) and infra-red (IR) wavelengths and produce a more natural image. The images were saved and serve as an additional data qualifier for properties of the radiation field at the time of measurement, e.g., detecting cloud cover.

#### 2.1.6. UBX-G7020 GPS

The geographic position, altitude, and time of each observation was obtained and recorded with every measurement. This information was used for geo-referencing satellite albedo measurements and to verify the height above ground level of each measurement. The GPS time was used in addition to the real-time clock on the Teensy.

#### 2.1.7. APC220 Radio

Radios on both devices were used to establish two-way communication between the payload and the ground control unit. The ground control unit was used to initiate a measurement. The measuring device onboard the UAS sends measurements to the ground for display to evaluate operations in near real time. 

#### 2.1.8. Nokia Screen

Periodic updates of each measurement were printed to a screen on the hand-held unit and resulting albedo was plotted after each measurement. 

#### 2.1.9. MLX90614 Infra-Red (IR) Sensor

The onboard IR thermometer faces downward to capture noncontact measurements of surface temperature with a temperature range from −70 °C to 380 °C and a temperature accuracy of ±0.5 °C. The detector has a field of view of approximately 100° with a peak zone around 0° where the measured value is the average temperature of all objects in the field of view.

#### 2.1.10. SD Card

All data parameters and camera images were saved to a 2-GB SD card. The SD port is built into the Teensy 3.6 microcontroller.

#### 2.1.11. Real-Time Clock

The real-time clock is built-in to the Teensy 3.6 microcontroller and was used for recording the time at which each measurement was taken. It is manually set once upon installation and reports both time and date.

### 2.2. System Calibration and Testing

#### 2.2.1. Diffuser Transmissivity

**Figure 3:**
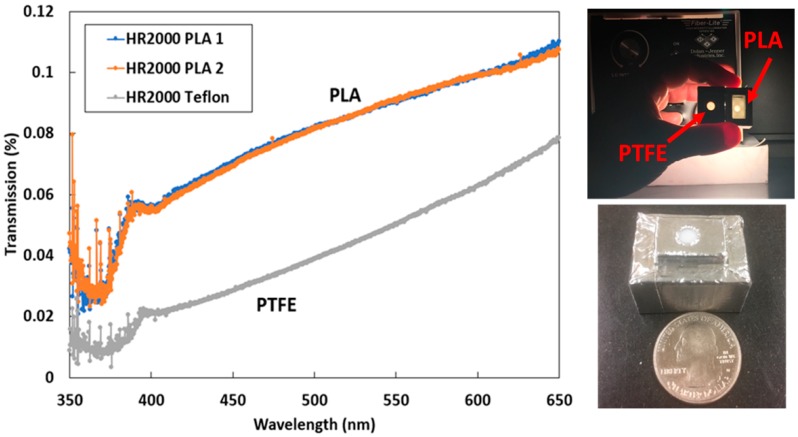
Transmissivity of polylactic acid (PLA) and Teflon diffusers using Ocean Optics HR2000 spectrometer. Both types of diffusers allowed very little light through (<1%). PLA was incorporated into the instrument design over polytetrafluoroethylene (PTFE) for ease of manufacture of the custom component by 3D printing and appropriate transmission for spectrometer integration time. The transmissivity decreases rapidly below 400 nm and for this reason we chose to limit the spectral range of our results to 400 nm.

To control the amount of solar radiation entering the detector, diffusers were 3D printed and fitted to each spectrometer. The diffusers were characterized for their transmissivity, angular response, and fluorescence. The transmissivity of the 3D-printed diffusers was tested using an Ocean Optics HR2000 spectrometer. Overall the diffusers allow 0.1% of light through and even less below 400 nm, as shown in Figure 3. It was found that below 400 nm the diffusers let in very little light and because of this our study only focuses on 400 nm and above. Additional motivation for characterizing the transmissivity of the 3D-printed diffusers was to check for any unwanted fluorescence. It was found that certain types of PLA fluoresced, however the final PLA diffusers used in the instrument design showed no signs of fluorescence. This experiment demonstrated that the PLA diffuser spectral variation was similar to commonly used polytetrafluoroethylene (PTFE) diffusers.

#### 2.2.2. Angular Response

**Figure 4:**
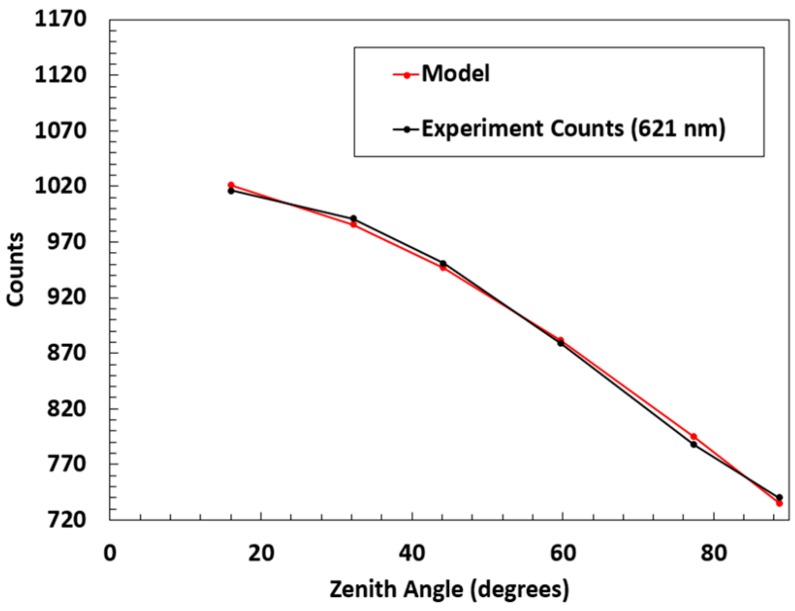
The cosine response of the instrument was measured to ensure proper use as an irradiance detector. Raw counts (no dark counts subtracted) from the spectrometer at 621 nm were recorded while tilting the detector every few degrees. The model curve includes an offset for the dark counts.

Albedo is measured as the hemispherical reflectivity of a surface as a function of wavelength [24]; therefore, an ideal albedo measuring device must have a reasonable cosine response and be multispectral. An experiment to test the cosine weighting of the instrument was performed using a light source and a lens to focus the light evenly onto the detector. The onboard absolute orientation sensor was used to record the zenith angle and measurements were taken in ~5° steps through controlled tilting of the instrument. Overall, the experimental measurements and the model cosine response are in accord, as shown in Figure 4, where the model cosine response is a cosine curve with amplitude appropriate to overlap with measurements.

#### 2.2.3. Temperature Compensation

An experiment to model the dark counts of the spectrometers was performed using two environmental chambers: a toaster oven and a freezer. The spectrometer, along with a temperature sensor, were breadboarded and subjected to extreme operating temperatures. A second-degree polynomial fit was taken from the resulting curve and equations for modeling the dark counts with respect to temperature in degrees centigrade were found for each spectrometer (Equations (3) and (4)). Temperature inputs for Equations (3) and (4) (*temp*) are obtained from the onboard pressure, temperature, and humidity sensor (Bosch model BME 280).

(3) $$Modeled\;Dark_{spectrometer1}=\;0.011\times \left(temp^{2}\right)+0.062\times temp+720\;$$

(4) $$Modeled\;Dark_{spectrometer2}=\;0.011\times \left(temp^{2}\right)+0.063\times temp+727\;$$

**Figure 5:**
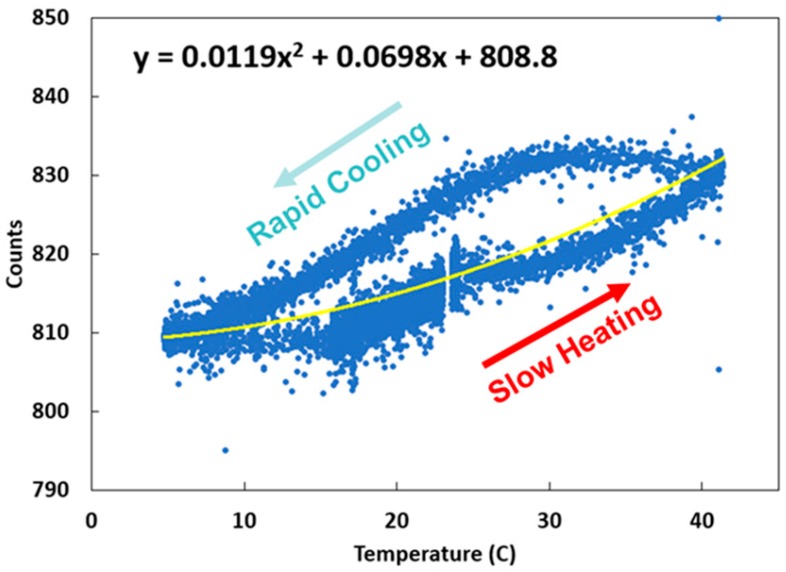
The two spectrometers onboard the instrument were tested and corrected for their temperature dependence of the dark counts (*y*-axis). The hysteresis curve is a result of the temperature measurements and the spectrometer counts not changing at the same rate. A second-degree polynomial fit was taken from the resulting curve, and equations for modeling the dark counts of the spectrometer with respect to temperature were derived. This is done to provide the dark counts while the instrument is flying.

The dark count uncertainty range was found to be approximately +/− 15 counts over the entire temperature range studied, where typical total counts under illumination are around 6000. The same procedure was done for each spectrometer, and it was found that dark counts from the two spectrometers differed by less than 10 counts. Experimental analysis was performed such that the dark counts were averaged over all the wavelengths. Differences in the response times of the temperature sensor and the spectrometer caused the resulting hysteresis curve, as shown in Figure 5. Discrepancies in the results could be due to the fact that the temperature sensor was not in direct contact with the spectrometer, and therefore did not represent the spectrometer actual temperature but instead the environmental temperature. Per manufacturer recommendations and to avoid condensation, we did not test below 5 °C.

#### 2.2.4. Transfer Function

A transfer function was calculated to correct for the differences between the two spectrometers and the slight variation in their diffusers (Equation (5)). Sensor counts with the dark counts subtracted from each spectrometer were used for calculation in Equation (5). The instrument was carefully flipped to obtain an upward and downward facing measurement for each spectrometer.

(5) $$H\left(\lambda \right)=\;\sqrt{\frac{Spec_{2}\;Downward}{Spec_{1\;}Downward}\times \frac{Spec_{2}\;Upward}{Spec_{1}\;Upward}}\;$$

**Figure 6:**
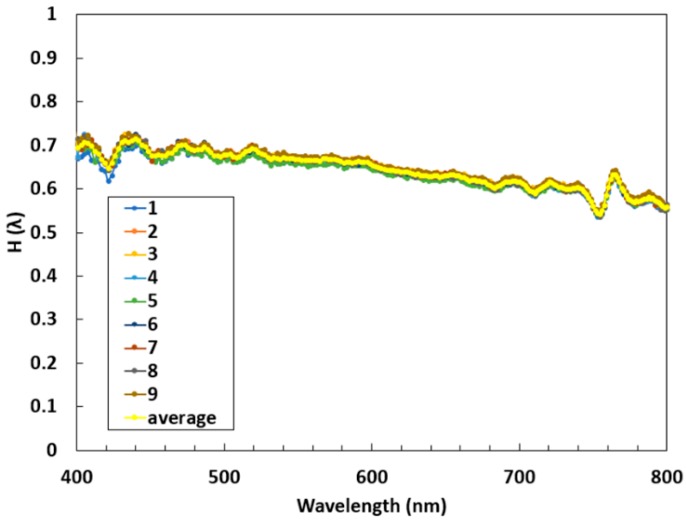
A transfer function to account for the differences in the two micro-spectrometers was calculated. Multiple measurements were taken over the same surface (right) and the average was applied to one spectrometer in order to “equal” the other.

This was done by taking multiple measurements over the same surface. Nine measurements taken over a grass and concrete covered area were averaged for each wavelength, as shown in Figure 6. The average was then applied to the output of one spectrometer to equal the other when measuring the same irradiance.

Final albedo is calculated according to Equation (6), where dark counts (*Dark*) are subtracted from spectrometer counts (*Spec*_1_, *Spec*_2_) and normalized by the integration time (*int time*). The transfer function (*H*) is then applied to the upward facing spectrometer (*Spec*_1_).

(6) $$Albedo\;\left(\lambda \right)=\;\frac{\frac{Spec_{2}\;-\;Dark\;}{int\;time}}{\left(\frac{Spec_{1}\;-\;Dark}{int\;time}\right)\;\times \;H\left(\lambda \right)}\;$$

#### 2.2.5. Preliminary Experiments

**Figure 7:**
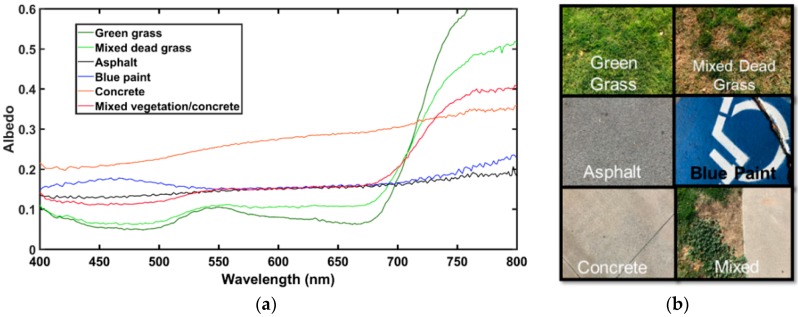
Initial testing of the instrument was performed over various surfaces around the University of Nevada, Reno (UNR) campus. The observed spectral signatures (**a**) align with expected signatures for the examined surface types (**b**). The data collected here were obtained using the instrument in the handheld version.

Initial testing of the instrument was performed over heterogeneous surfaces around the University of Nevada, Reno (UNR) campus. Surface reflectance values for common surface types were examined: vegetation, dead vegetation, concrete, asphalt, blue paint, and mixed vegetation, as shown in Figure 7. For verification, the results were qualitatively compared with the United States Geological Survey (USGS) online spectral library. Overall the instrument performed well and produced appropriate spectral signatures for vegetation, blue paint, asphalt, and concrete.

## 3. Methods 

### 3.1. Nevada Black Rock Desert Experiment

**Figure 8:**
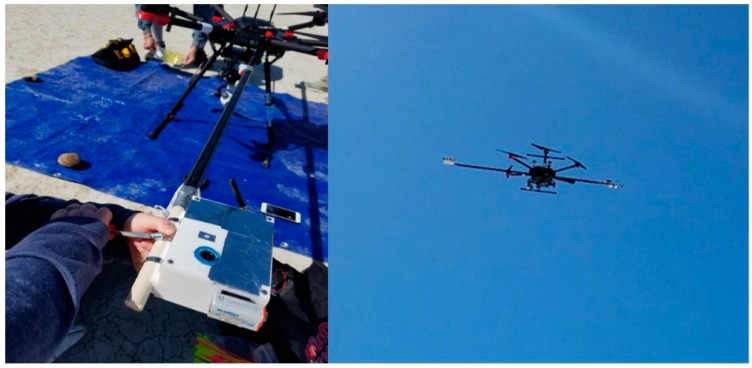
Configuration of instrument mounted to unmanned aircraft system (UAS). A long pole, approximately 2 m in length, was used to extend the instrument away from the body of the aircraft. This was done to limit the effects of the aircraft on albedo measurements, specifically those that would change the surrounding radiation field.

The instrument was deployed in Nevada’s BRD under clear sky conditions on 5 October 2017. The homogeneous terrain of the BRD was chosen as our study location for its known high surface reflectance. The surface of the BRD is representative of other areas known to also have a high surface reflectance in the western U.S. To obtain albedo, the measuring device was mounted onto a hexacopter UAS (DJI model Matrice 600 Pro). The rotary wing aircraft has dimensions 525 × 480 × 640 mm with a total weight (including batteries) of 10 kg and a recommended payload weight of 5.5 kg. With all payload attachments onboard, the aircraft could fly for approximately 20 min. The instrument was mounted onto a carbon fiber pole that extended out from the aircraft to limit the aircraft’s influence on the radiation field, as shown in Figure 8. The aircraft was manually piloted over two surface types at four heights above ground level (AGL): 30.5 m, 61.0 m, 91.4 m, and 119.8 m, to simulate the spatial sensing area of sensors on satellites. At 30.5 m AGL the ground field of view matches the 500 m spatial resolution of MODIS with a detector field of view of ~166°, as shown in Appendix A. Multiple measurement heights were used to explore the variability in the albedo measurements at different heights and assess the performance of the overall system. The heights were approximately 30 m height increments up to the maximum allowable UAS flight height of 119.8 m AGL. Over 90% of the measured signal is received with an 80° instantaneous field of view (IFOV) and was therefore assumed for the ground IFOV calculation. Flights were made as close to solar noon as possible, and during flights the instrument was oriented to face the sun to avoid disturbances to the radiation field due to shadowing.

**Figure 9:**
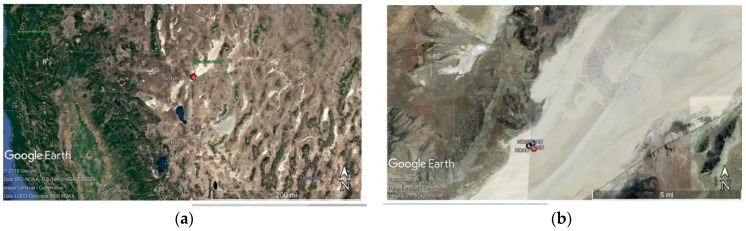

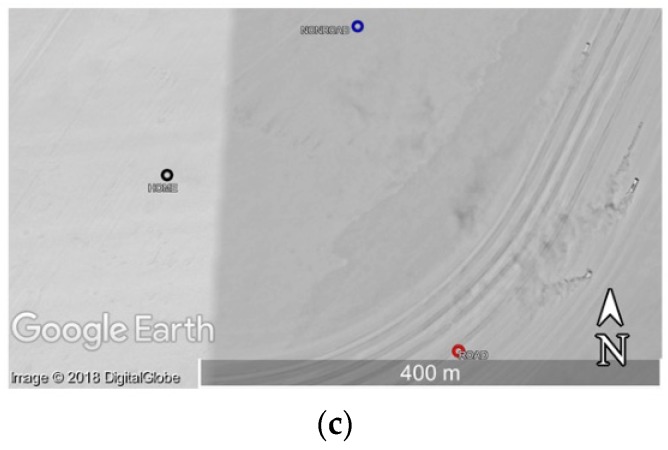
Field site locations: (**a**) Overview of Black Rock Desert (BRD) located north of Reno. (**b**) Zoomed-in Google Earth image over the BRD showing proximity to annual Burning Man Festival (the half circle). (**c**) Zoomed in Google Earth image over the location where measurements were made. The blue circle (most north) represents “non-road” (40.749586, −119.261153), the red circle (most south) represents “road” (40.748192, −119.258969) and the black circle in the middle represents the location of the UAS pilot (40.748345, 119.263186).

Measurements were obtained over two locations, designated in Figure 9, as the red and blue circles for road and non-road, respectively. The physical appearance of the two locations varied, however their compositions were believed to be similar. In one location, denoted “road”, the ground surface had been consistently driven over as a means in and out of the yearly Burning Man event, which had taken place the previous month. The “non-road” location showed less evidence of vehicle tracks and showed no distinct disturbance from car tracks. In other words, the tracks appearing over the location of the non-road observations were sparse and random compared to the road location. Five measurements were obtained at four different heights above each road and non-road location. Per Federal Aviation Administration (FAA) Part 107 regulation, our flight height was restrained to below 400 feet (121.9 m).

### 3.2. Satellite Remote Sensing Products

MODIS instruments onboard NASA’s Terra (morning overpass) and Aqua (afternoon overpass) satellites collect global atmospheric measurements. The twin MODIS instruments have been used to study atmospheric processes, air quality, and factors which impact the earth’s radiative budget [25,26]. Terra and Aqua daily surface spectral reflectance retrievals [bands 3 (459–479 nm), 4 (545–565 nm), and 1 (620–670 nm)] at 250 m (Band 1) and 500 m (Band 3 and 4) horizontal resolution were used to compare with the multispectral albedometer measurements [27]. In addition, LANDSAT7 ETM+ instrument obtains surface albedo values with higher horizontal resolution (30 m) than the MODIS albedo products. The spectral bands used in this evaluation are 450–520 nm, 520–600 nm, 630–690 nm, and 770–900 nm [28]. 

MODIS aerosol optical properties from the enhanced deep-blue (DB) algorithm from collection 6.1 were used to study the impact of surface reflectance on *AOD* retrievals at 10 × 10 km horizontal resolution. The DB algorithm uses a data base of surface reflectivity, a dynamic surface reflectance, a normalized difference vegetation index, and a radiative transfer model that tracks polarization for select locations to better retrieve *AOD* over bright surfaces [29,30]. Modifications in the collection 6.1 algorithm provide better capture of fire plumes and a better characterization of *AOD* over bright surfaces compared to previous algorithm versions (i.e., collection 6) [16]. The DB algorithm offers greater spatial coverage than other satellite derived algorithms for aerosol optical properties (e.g., dark-target) because desert surfaces (e.g., BRD) are dimmer at short wavelengths [14,29,31,32,33]. Two DB *AOD* products were used in this investigation the Deep_Blue_Aerosol_Optical_Depth_550 Land (DB) for all quality flags (0, 1, 2, and 3) and Deep_Blue_Aerosol_Optical_Depth_550_ Land_Best_Estimate (DB-Best) for high quality flags (2 and 3). The expected error of the DB *AOD* product (550 nm) over land is expected to be ±0.03 + 0.21 × *AOD* for arid path retrievals and ±0.03 + 0.18 × *AOD* for vegetated path retrievals for collection 6.1 [34].

DB spectral *AOD* land and spectral single scattering albedo land (SSA or $\tilde{\omega }$) products (412, 470, and 660 nm) were used to estimate the deviation of the spectral slope of retrieved *AOD* due to surface reflectance. The asymmetry parameter was assumed to be 0.73 for short wavelength (412, 470 nm) and ~0.71 for the 660 nm channel for low absorbing dust particles [35]. The method used to spatially average *AOD* and create the maps for DB (quality flags of 1, 2, and 3) is explained by Loría-Salazar et al. (2016) [14]. The horizontal domain for California and Nevada ranged from 127° W to 114° W longitude and from 32° N to 42.5° N latitude, and for the BRD 119.4° W to 118.5° W longitude and 40.6° N to 41.5° N latitude. The temporal domain was October 2017.

## 4. Results

### 4.1. Albedo

Comparisons of UAS and satellite retrieved surface reflectance values over Nevada’s BRD are presented here to understand the impacts of heterogeneous land surfaces on albedo. Five albedometer measurements at each height AGL were averaged and compared to single pixel values from MODIS and LANDSAT7 ETM+. For MODIS, the pixel value for road and non-road areas were the same due to the large spatial resolution (250 m for band 1 and 500 m for bands 3 and 4). The higher spatial resolution (30 m) of LANDSAT7 ETM+ allowed for the pixels to be categorized by road and non-road areas to compare with the road and non-road areas measured using the UAS.

**Figure 10:**
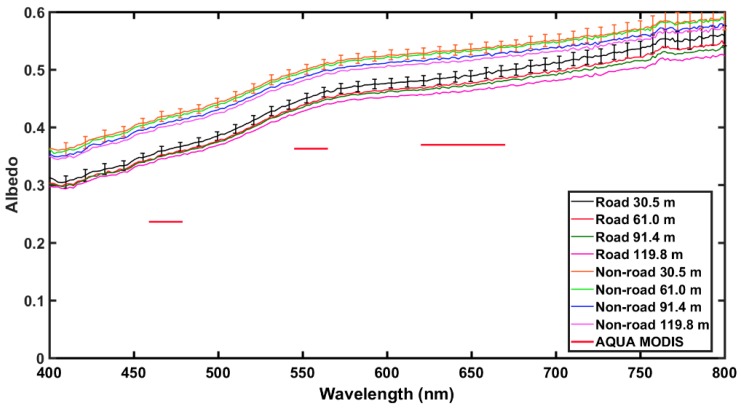
Albedometer measurements obtained over Nevada’s Black Rock Desert on 5 October 2017 at ~21:00 UTC and comparison to Aqua MODIS (Moderate Resolution Imaging Spectroradiometer) retrieved surface reflectance. Plotted are the average of five measurements taken at each height above ground level (AGL) over road and non-road. Uncertainty in the albedometer measurements was calculated according to Equation (2) and displayed for only two heights to prevent overcrowding in the figure. The mean uncertainty was ~0.01 across all wavelengths for each measurement across all heights.

**Figure 11:**
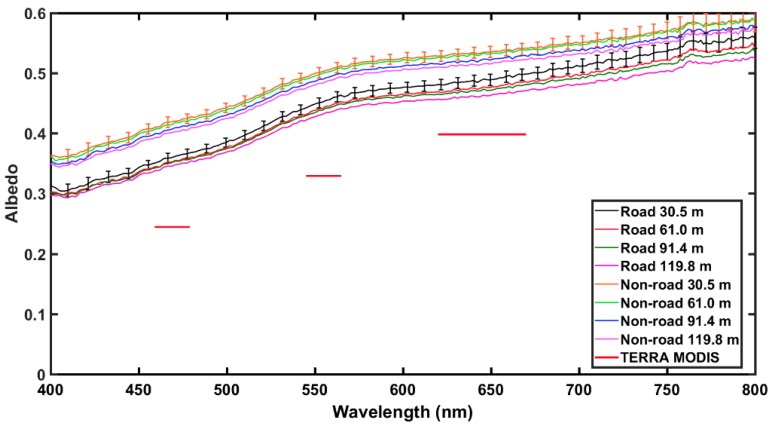
Same caption as Figure 10 but for Terra MODIS.

**Figure 12:**
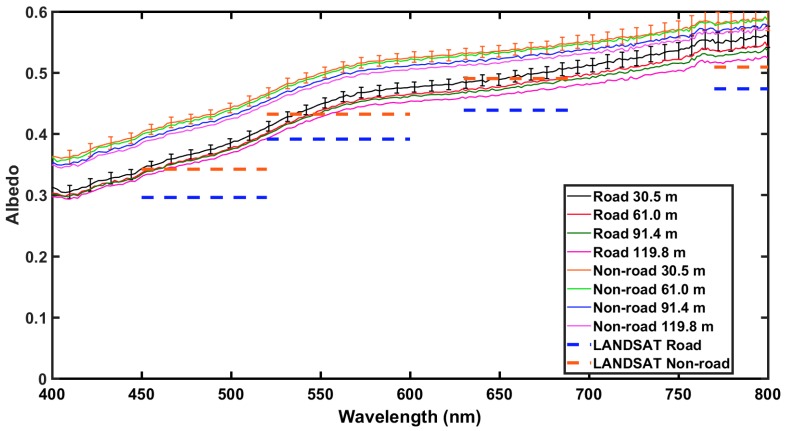
Same caption as Figure 10 and Figure 11 but for Land Satellite 7 (LANDSAT7) Enhanced Thematic Mapper Plus (ETM+).

In general, both the satellite and albedometer non-road measurements exhibited a higher albedo than those obtained over the road location; likely due to shadows caused by ridges created by vehicle tracks. Measured albedo over non-road locations ranged from ~0.35 at 400 nm to ~0.60 at 750 nm and from ~0.30 at 400 nm to ~0.50 at 750 nm over road locations, as shown in Figure 10, Figure 11 and Figure 12. Over both road and non-road locations albedo tended to decrease with increasing height AGL. In other words, albedo measurements made closer to the surface were slightly greater than those made tens of meters above the surface. This can likely be attributed to the differences in the amount of atmosphere present between the albedometer and the surface. At greater heights AGL, there is more atmosphere to contribute to the extinction of reflected shortwave radiation. Additionally, at greater heights the detector is sensing over a larger spatial area that could contribute to the overall variability in the measurements. The observed range in measured albedo from the lowest height to the highest height (30.5 m to 119.8 m) was within 0.05 over road and non-road locations, indicating that the effect of height AGL was less than the effect of road versus non-road location. 

In comparison to Aqua MODIS, measurements obtained with the albedometer were higher across all MODIS bands, with differences of ~26% averaged over road and ~34% averaged over non-road areas, as shown in Figure 10. A similar trend was observed for comparison to Terra MODIS, with all bands lower than albedometer values by approximately 26% for road and 34% for non-road, as shown in Figure 11. When compared to MODIS 8-day best values, the measured albedometer values compared better to the MODIS 8-day best values than daily MODIS retrievals (figure not shown). In comparison to LANDSAT7 ETM+, measurements obtained with the albedometer were again higher across all bands, as shown in Figure 12. However, LANDSAT7 ETM+ values were closer to albedometer measured values than MODIS (percent difference ~15% over road and ~14% over non-road), likely due to the enhanced spatial resolution. Another important difference to note is that the albedometer obtains a scene albedo, while the satellites obtain a surface albedo within a specific footprint.

The albedometer accuracy depends on careful determination of the transfer function (Equation (6)) immediately prior to albedo measurements. Ensuring the levelness of the detector is left up to the hovering capabilities of the aircraft and accurate measurements require carefully mounting the instrument level to the aircraft and reducing instrument vibrations as much as possible. In 3D printing the diffusers, the angular response needs to be measured to ensure that different printing patterns do not compromise the cosine response of the detector. Flight heights made closer to the surface are best for comparison to satellite retrievals due to the large ground field of view of the irradiance detector, as shown in Appendix A.

**Figure 13:**
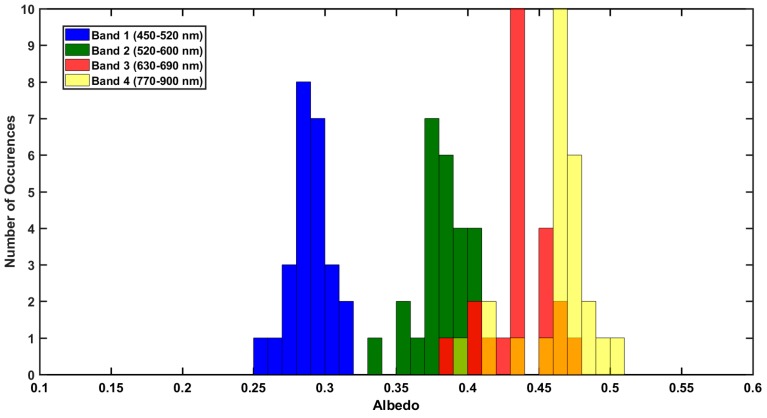
Histogram of neighboring LANDSAT7 ETM+ pixels over road location on 5 October 2017.

**Figure 14:**
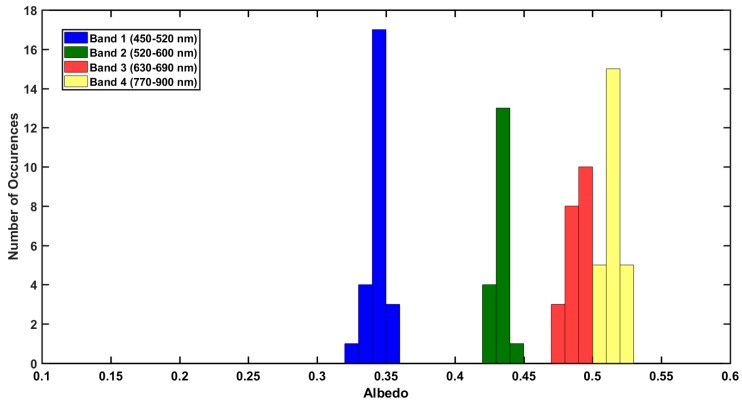
Histogram of neighboring LANDSAT7 ETM+ pixels over non-road location on 5 October 2017.

To investigate the variability in the satellite products, histograms were generated for neighboring LANDSAT7 ETM+ pixels around road and non-road locations, as shown in Figure 13 and Figure 14. LANDSAT7 ETM+ values were generally found to be near albedometer measurements for both road and non-road locations. However, there appears to be a wider range of albedo values over the road location compared to the non-road location which implies a greater variation in the road surface. Measured albedo from the UAS were in the range of neighboring pixel values for LANDSAT7 ETM+. Histograms of neighboring pixels for MODIS were not used due to the low spatial resolution of the sensor.

**Figure 15:**
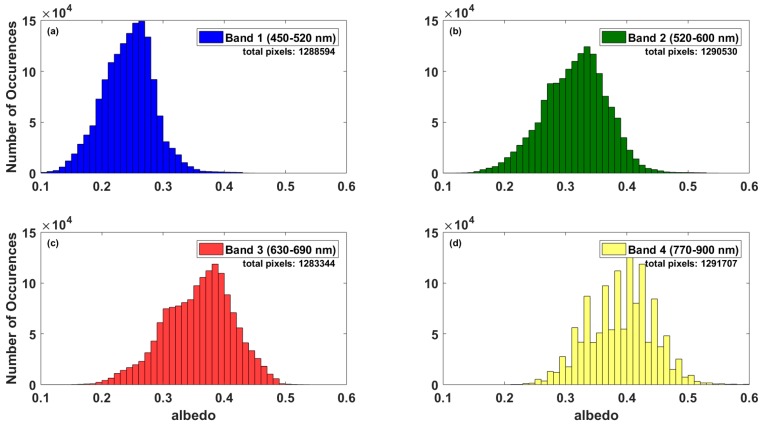
Histogram of all LANDSAT7 ETM+ pixels over Nevada’s Black Rock Desert on 5 October 2017. (**a**) Band 1 (450–520 nm). (**b**) Band 2 (520–600 nm). (**c**) Band 3 (630–690 nm). (**d**) Band 4 (770–900 nm).

In addition to neighboring pixels, a histogram of pixel values across the entire BRD was made to assess the homogeneity of the desert surface. All valid pixels in the region of interest are incorporated into the histogram in Figure 15, excluding missing data and over saturated pixels. The histogram had a wide range of albedo values across the entire desert for bands 1, 2, and 3, while band 4 albedo values were mostly within the 0.4–0.5 range. This indicates that the Black Rock Desert is heterogeneously bright. The more consistent grouping of observed pixel values at band 4 is likely representative of a common surface type known to be present in dry lake beds such as the BRD. The saline minerals present in playas exhibit absorption features in the near-infrared bands, and their reflectance in the visible-near infrared (VNIR) is highly dependent on moisture content [36]. The spread of albedo values obtained from LANDSAT7 ETM+ across the BRD is likely a consequence of some parts being more wet than others for at least certain parts of the year. Brightness of the BRD playa is therefore likely to be sensitive to weather patterns and seasonal variation.

### 4.2. Impact of Surface Albedo on Satellite AOD Retrievals

**Figure 16:**
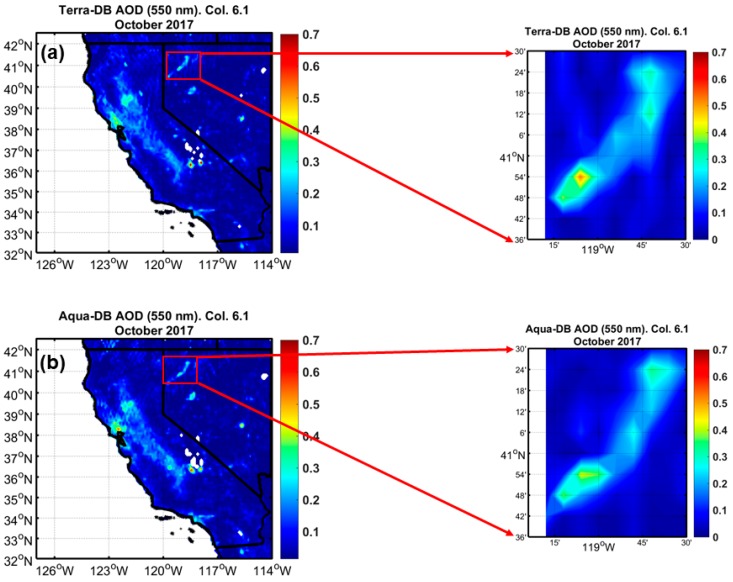
Monthly averages during October 2017 of MODIS collection 6.1 deep-blue (DB) aerosol optical depth (AOD) (550 nm) over California and Nevada, with the Black Rock Desert in the zoomed image: (**a**) Terra DB AOD. (**b**) Aqua DB AOD.

Figure 16 shows monthly averages of *AOD* (550 nm) DB during October 2017. High *AOD* values (*AOD* from ~0.3 to ~0.4) located at the Bay Area in California were due to a local fire (visual inspection from NASA World View). Because of the high aerosol signal over the fire, the DB algorithm shows high quality data. However, over surfaces with high surface reflectance (e.g., BRD), the DB algorithm was not able to adequately retrieve *AOD* and the retrievals are low quality. Therefore, those pixels were removed from the highest quality product (DB-best). Because of the impact of high surface reflectance, the *AOD* values over the BRD are unrealistically high. These results were also found by Loría-Salazar et al. (2016) [14]. Based on this, satellite retrievals of *AOD* are facing two major limitations over regions with high surface reflectance: (1) Unrealistically high *AOD* values using low quality data, as shown in Figure 16a,b, and (2) lack of data points (zero retrievals) if high quality data is used. While the developers of the DB algorithm stress the importance of only using the high quality (DB-best) product it is important to do research investigations to help determine the physical processes causing these high *AOD* values to inform future algorithm improvements. The limitations that occur in regions with high surface reflectance impede the study of the mineral dust aerosol that tends to occur over bright, arid surfaces. Over the BRD, the high *AOD* fingerprint is due to surface reflectance issues in the algorithm that causes the *AOD* values to be unrealistically high.

To further investigate the impact of surface reflectance in the *AOD* retrievals, a 1-D radiative model was used to calculate the deviation of *AOD* due to the change in surface reflectance, as shown in Appendix B. The aim of using this simple analytical model is to provide a calculation for the uncertainty in *AOD* with respect to surface albedo and provide a qualitative basis for the importance of improving surface reflectance values in satellite *AOD* retrieval algorithms. The deviation of *AOD* due to the change in surface reflectance is calculated using Equation (7):

(7) $$\frac{\partial AOD}{\partial A}\sim \frac{1}{\left[2A\left(1-\frac{\tilde{\omega }\left(1+g\right)}{2}\right)\;-\;\frac{\tilde{\omega }\left(1-g\right)}{2}\right]}\;.$$

Here, the single scattering albedo was taken from the DB algorithm ($\tilde{\omega }$~0.975 for 660 nm), *g* is the asymmetry parameter (*g*~0.71 for 660 nm) for weakly-absorbing dust [35], and *A* is surface albedo taken from the UAS measurements discussed above (*A*~0.48 for 660 nm). This simplified 1-D model is applicable when (*AOD* < 0.1). It is important to note here that a full investigation of the uncertainty in *AOD* with respect to the single scattering albedo and asymmetry parameter would be required to improve the *AOD* retrieval algorithms, however it is outside the scope of this paper. A discussion of Equation (7), including model assumptions and the physical interpretation, is given in Appendix B. For both Terra and Aqua retrievals on 5 October 2017 and monthly averages, the sensitivity of *AOD* due to albedo is ∂AOD/∂A~ 54.5 for 660 nm. This result implies that *AOD* satellite retrievals over bright surfaces like the BRD are extremely sensitive to errors in surface albedo but also in single scattering albedo values. Retrieving different aerosol types, for example bright (dust) versus dark (black carbon), over different surfaces such as deserts (bright) versus oceans (dark) complicates the process of retrieving *AOD*. Therefore, more accurate measurements of surface reflectance and the development of more detailed land cover data sets are required to improve *AOD* retrievals over bright surfaces, especially in the semi-arid western U.S. Finally, the deviation of *AOD* due to albedo, *SSA*, and *g* parameters must also be considered in future research to study the sensitivity of each parameter on the retrieved *AOD*. 

## 5. Discussion

The instrument development goal of this research was to develop a lightweight multispectral instrument to measure albedo. The primary application was to measure the albedo of a representatively bright area of the Black Rock Desert for comparison with albedo from satellite remote sensing, and ultimately to collect better surface reflectance data to address issues with aerosol optical depth retrievals from sensors onboard satellites. Our albedometer also serves as a general tool for quantifying surface albedo at a variety of time and length scales. It has broad applications for use in climate research and environmental monitoring. The instrument is an inexpensive alternative to existing commercial devices and could serve as a valuable addition to field instrumentation. The design of the instrument allows for measuring the albedo of glaciers in mountaineering environments, for airborne measurements from aircraft, and for ground-based measurements over complex terrain.

The instrument was flown onboard a UAS to introduce a novel technique for making airborne albedo measurements. We demonstrate that it is possible to accurately measure albedo at low altitudes using a UAS. Slight increases in albedo with decreasing height AGL indicate the need for possibly applying an atmospheric correction to measurements even when flown a few hundred feet above the surface, and for interpretation of the effective field of view of the instrument. Use of a UAS provides for obtaining albedo measurements over a large spatial area at a much lower cost compared to using a high-altitude aircraft without compromising accuracy of results.

We deployed the instrument in a desert environment to compare our measurements against satellite retrieved surface albedo. MODIS and LANDSAT7 ETM+ surface reflectance consistently underestimated measured albedo across all spectral bands. It was found that LANDSAT7 ETM+ retrieved values agreed more with measured albedo values than MODIS did, likely due to the finer spatial resolution of the LANDSAT7 ETM+ instrument. The underestimation of satellite retrieved surface reflectance likely contributes to an overestimation in observed aerosol optical depth from satellite remote sensing. 

The discrepancies observed between ground-based and satellite-retrieved *AOD* in the western U.S. are likely due to the underestimation of surface reflectance over heterogeneous surfaces [14,16]. Similar results were found in Lahore and Karachi, Pakistan [37], and Alberta, Canada [38], where elevated surface reflectance diminished the accuracy of AOD satellite retrievals. The DB algorithm from collection 6.1 is the recommended MODIS aerosol product for use over bright surfaces. However, it still presents limitations over highly reflective surfaces (e.g., the BRD). The low-quality product from DB overestimated *AOD* and the high-quality product from DB removed the entire desert inhibiting the understanding of the impact of dust in air quality, radiative transfer, and atmospheric processes studies. Results from this investigation showed that *AOD* retrievals are highly impacted by surface reflectance. Therefore, increasing the number of accurate measurements of surface albedo and an intensive evaluation of albedo satellite retrievals are required to improve aerosol satellite retrievals. 

Future work could consist of developing the instrument for radiance measurements as well as for Bidirectional Reflectance Distribution Function (BRDF) measurements. Efforts to increase the wavelength range of the instrument would provide more thorough albedo measurements. Additional measurements over the entire Black Rock Desert are needed for a more comprehensive data set for comparison to satellite retrievals, as well as expanding the study to other areas known to have a high surface reflectance in the western U.S. Additional comparison to other airborne platforms, such as manned aircraft for measuring surface reflectance, could also be explored. Analysis of seasonal variations in albedo over the Black Rock Desert could be useful for improving satellite retrievals of *AOD* and for general climate studies. Incorporating ground-based sun photometer measurements collocated with albedo measurements would provide additional comparison to evaluate satellite retrievals. Overall, the instrument has potential to be transformed into a stationary device and could be designed to be mounted onto a tower with a more weather-proof case and a long-term power supply. 

## Figures and Tables

**Figure A1:**
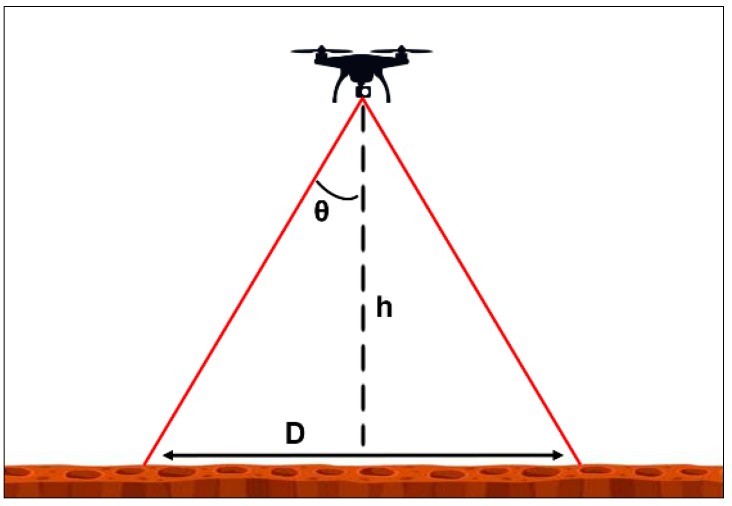
Diagram of the detector field of view while flying. A change in height (*h*) above the ground level will affect the spatial area that the instrument senses at the surface (*D*).

## Abbreviations

AGL	above ground level
AOD	aerosol optical depth
ARM	advanced reduced instruction set computer machine
BRD	Black Rock Desert
BRDF	Bidirectional Reflectance Distribution Function
CMOS	complementary metal-oxide-semiconductor
DB	deep-blue
DOE	Department of Energy
ETM+	Enhanced Thematic Mapper Plus
FAA	Federal Aviation Administration
GIFOV	ground instantaneous field of view
GPS	Global Positioning System
I2C	inter-integrated circuit
IDE	Integrated Development Environment
IFOV	instantaneous field of view
IR	infra-red
LANDSAT7	Land Satellite 7
MODIS	Moderate Resolution Imaging Spectroradiometer
NASA	National Aeronautics and Space Administration
NOAA	National Oceanic and Atmospheric Administration
PLA	polylactic acid
PTFE	polytetrafluoroethylene
SD	secure digital
SSA	single scattering albedo
UAS	unmanned aircraft systems
UNR	University of Nevada, Reno
U.S.	United States
USGS	United States Geological Survey
UV	ultraviolet
VNIR	visible-near infrared

## Appendix A

Approximation of ground instantaneous field of view (GIFOV)

(A1) D≈2htan(θ). 

In Equation (A1), *D* is the ground field of view, *h* is the height above ground level, and *2θ* is the angular field of view of the detector.

## Appendix B

To further investigate the impact of surface reflectance on the *AOD* retrievals, a 1-D radiative model was used to calculate the sensitivity of *AOD* to surface reflectance. The purpose of this simple model is to provide an analytical expression that can be used for understanding the sensitivity of *AOD* retrievals to the surface albedo using a propagation of error approach to quantify uncertainty [39]. The analytical approach is to obtain $\frac{\partial AOD}{\partial A}$ so that in propagation of error we may obtain a measure of the uncertainty $\Delta AOD=\Delta A\;\left[\frac{\partial AOD}{\partial A}\right]$ as it relates to the uncertainty in surface albedo ΔA. The question is, how do uncertainties in surface albedo measurement propagate into uncertainties in aerosol optical depth? Because the focus here is to understand the impact of surface reflectance on *AOD* retrievals, a partial derivative is used to quantify the uncertainty. To obtain the partial derivative, the assumptions are that the aerosol layer is infinitesimally thin, and that for 660 nm both Rayleigh (~0.04) and aerosol optical depth (<0.1) are much less than unity so that single scattering and single surface reflection apply. The total radiation received by the satellite is defined by

(A2) $$\;I_{Total}=I_{1}+I_{2}.\;$$

Here, *I*_1_ is the direct beam radiation reflected by the surface, and *I*_2_ is the diffusely scattered radiation due to back and forward scattering from the presence of aerosol in the atmosphere. *I_1_* is given by

(A3) $$I_{1}=I_{0}e^{-2AOD}A\approx I_{0}\left(1-2AOD\right)A.$$

Here, *I*_0_ is the top of the atmosphere solar irradiance and *A* is the surface albedo. The aerosol layer is taken to backscatter and forward scatter with strength

(A4) $$I_{2}=I_{0}AOD\tilde{\omega }\frac{\left(1-g\right)}{2}+2\;I_{0}A\;AOD\tilde{\omega }\frac{\left(1+g\right)}{2}\;.$$

The incident beam reflects off the surface (Equation (A4)) and is attenuated and forward scattered twice by the aerosol layer with the assumption of single scattering and single surface reflection. Here $\tilde{\omega }$ is the single scattering albedo and *g* is the asymmetry parameter.

To proceed, we solve Equations (A2)–(A4) for *AOD*. To that end, we first define γ as

(A5) $$\;\gamma \equiv \frac{I_{Total}}{I_{0}}=AOD\left[\frac{\tilde{\omega }\left(1-g\right)}{2}+A\left(\tilde{\omega }\left(1+g\right)-2\right)\right]+A,\;$$

and solve for *AOD*:

(A6) $$\;AOD=\frac{\gamma -A}{\left[\frac{\tilde{\omega }\left(1-g\right)}{2}+A\left(\tilde{\omega }\left(1+g\right)-2\right)\right]}\;.$$

To analytically determine the uncertainty of *AOD* with respect to *A* using propagation of error the partial derivative is used. Taking the partial derivative of *AOD* (Equation (A6)) with respect to *A,* and using Equation (A5) for γ gives

(A7) $$\frac{\partial AOD}{\partial A}\sim \frac{1-2AOD\;\left[1-\frac{\tilde{\omega }\left(1+g\right)}{2}\right]}{\left[2A\left(1-\frac{\tilde{\omega }\left(1+g\right)}{2}\right)\;-\;\frac{\tilde{\omega }\left(1-g\right)}{2}\right]}.$$

In the limit of small *AOD* (i.e., AOD≤0.1) this simplifies to

(A8) $$\frac{\partial AOD}{\partial A}\sim \frac{1}{\left[2A\left(1-\frac{\tilde{\omega }\left(1+g\right)}{2}\right)\;-\;\frac{\tilde{\omega }\left(1-g\right)}{2}\right]}.$$

Equation (A9) is the relationship used for determining the sensitivity of *AOD* retrievals to surface albedo and can be used as an approximation for the uncertainty of *AOD* estimates due to uncertainties in surface albedo.

To further illustrate the importance of surface albedo on *AOD* retrievals we look at the physical interpretation of Equation (A8). Looking at the limits of Equation (A8), note first that ∂AOD/∂A→∞ when the denominator goes to zero. Based on this, the denominator of Equation (A8) can be set equal to zero to find a critical value of surface albedo *A_crit_* “…defined as the surface albedo where the reflectance at top-of-atmosphere does not depend on *AOD*…” [40],

(A9) $$\;A_{crit}=\;\frac{1}{2}\frac{\tilde{\omega }\left(1-g\right)/2}{\left[1-\tilde{\omega }\;\left(1+g\right)/2\right]}\;.$$

The physical interpretation of Equation (A9) is that the backscatter by the aerosol layer is the same as the combination of forward scattering by the layer and backscattering by the surface albedo. To illustrate the critical albedo using Equation (A9) with $\tilde{\omega }=0.97$ and g=0.7 it can be found that $A_{crit}=0.42$. At and near this value of surface albedo, the uncertainty in *AOD* retrievals is very large. Interestingly, the sign of ∂AOD/∂A changes from negative to positive at $A_{crit}$ meaning that the uncertainty in surface albedo does not systemically lead to the same *AOD* uncertainty.

In another limit as $\tilde{\omega }\to 0$, then ∂AOD/∂A≈1/2A with the implication that detection of dark aerosol over a dark surface is impossible and the brighter the surface the more detectable the dark aerosol will be the smaller the error due to surface albedo. This was also found by Seidel and Popp (2012) “…we showed that the retrieval error is rather small for absorbing aerosols over bright surfaces” [40].
